# Data for long-term marginal abatement cost curves of non-CO_2_ greenhouse gases

**DOI:** 10.1016/j.dib.2019.104334

**Published:** 2019-07-29

**Authors:** Mathijs J.H.M. Harmsen, Detlef P. van Vuuren, Dali R. Nayak, Andries F. Hof, Lena Höglund-Isaksson, Paul L. Lucas, Jens B. Nielsen, Pete Smith, Elke Stehfest

**Affiliations:** a PBL Netherlands Environmental Assessment Agency, Bezuidenhoutseweg 30, NL-2594 AV, The Hague, the Netherlands; b Copernicus Institute of Sustainable Development, Utrecht University, Princetonlaan 8a, NL-3584 CB, Utrecht, the Netherlands; c Institute of Biological and Environmental Sciences, School of Biological Sciences, University of Aberdeen, 23 St Machar Drive, Aberdeen AB24 3UU, Scotland, UK; d Air Quality and Greenhouse Gases Program, International Institute for Applied Systems Analysis, A-2361 Laxenburg, Austria

**Keywords:** Non-CO_2_, Mitigation, MAC curves, Climate policy

## Abstract

This dataset represents long-term marginal abatement cost (MAC) curves of all major emission sources of non-CO_2_ greenhouse gases (GHGs); methane (CH_4_), nitrous oxide (N_2_O) and fluorinated gases (HFCs, PFCs and SF_6_). The work is based on existing short-term MAC curve datasets and recent literature on individual mitigation measures. The data represent a comprehensive set of MAC curves, covering all major non-CO_2_ emission sources for 26 aggregated world regions. They are suitable for long-term global mitigation scenario development, as dynamical elements (technological progress, removal of implementation barriers) are included. The data is related to the research article: “*Long-term marginal abatement cost curves of non-CO*_*2*_*greenhouse gases*” [1].

**Table:** Specifications Table

Subject	Environmental Science
Specific subject area	Climate Policy, GHG mitigation
Type of data	Table
How data were acquired	Literature review, in combination with model calculations with the IMAGE 3.0 integrated assessment model
Data format	Raw and Filtered
Parameters for data collection	The collected data is based on peer-reviewed studies of reduction potentials and costs of mitigation measures for the main non-CO_2_ greenhouse gas emission sources.
Description of data collection	The collected data represents emission reduction potentials and costs found in literature (based on both existing datasets and studies on individual reduction measures), converted into world region-specific marginal abatement costs curves for the main non-CO_2_ greenhouse gas emission sources.
Data source location	Institution: PBL Netherlands Environmental Assessment Agency City: The Hague Country: The Netherlands
Data accessibility	With the article
Related research article	Author's name: Mathijs J.H.M. Harmsen, Detlef P. van Vuuren, Dali R. Nayak, Andries F. Hof, Lena Höglund-Isaksson, Paul L. Lucas, Jens B. Nielsen, Pete Smith, Elke Stehfest Title: Long-term marginal abatement cost curves of non-CO_2_ greenhouse gases Journal: Environmental Science & Policy DOI: https://doi.org/10.1016/j.envsci.2019.05.013[1]

**Table:** 

**Value of the Data** • *Why are these data useful?* Updated estimates of long-term reduction potentials and costs of non-CO_2_ GHG emissions are crucial in climate policy research. These Non-CO_2_ MAC curves are based on the most recent insights in large body of literature. • *Who can benefit from these data?* These data are particularly beneficial for integrated assessment modellers who are involved in developing long-term climate policy scenarios. • *How can these data be used for further insights and development of experiments?* These more detailed estimates of long-term non-CO_2_ reduction potentials and costs can provide a valuable input in further global climate policy scenario development and assessments. • *What is the additional value of these data?* The data represent a comprehensive set of MAC curves, covering all major non-CO_2_ emission sources for 26 aggregated world regions. They are suitable for long-term global mitigation scenario development, as dynamical elements (technological progress, removal of implementation barriers) are included

## 1 Data

These documents contain CH_4_ and N_2_O (Data_MAC_CH4N2O_Harmsen et al._PBL) and fluorinated gas (Data_MAC_F-gases_Harmsen et al._PBL) marginal abatement cost (MAC) curves for all major global emission sources. Values represent relative emission reductions for the different emission sources at different marginal cost levels for the period 2015–2100 (not all intermediate years are provided, but values between subsequently provided years can be linearly interpolated). Two sets are made available: 1) One baseline-independent set with relative reductions compared to the global average emission factor in 2015 for the emission source concerned. Negative values represent a higher emission factor than the global average in 2015. 2) One set compatible with the IMAGE SSP2 baseline scenario (with “SSP2″ in the name of the sheet). Source-specific emission reductions in SSP2 are deducted from the reductions in the baseline-independent MACs. Implementation costs are provided in (2005/2010) $/tonne of C equivalents, assuming the use of the AR4 100 yr GWP potential.

## 2 Experimental design, materials, and methods

The MAC curves represent the combined reduction potential of all relevant mitigation measures at specific marginal costs for a specific emission source and country or region. In order to be relevant for long term climate policy projections, they account for future changes in reduction potential and costs, due to 1) technological learning and 2) removal of implementation barriers.

The MAC curves developed in this study are based on a combination of existing datasets and an assessment of individual mitigation options described in literature.

The reduction potential (*RP*) (in %) of a single mitigation measure in year *t* and region *r* is determined by:

(1) *RP*_(*t*,*r*)_ = *TA*_(*r*)_∗*RE*∗*IP*_(*t*)_∗*OVcorr*_(*t*,*r*)_

With (all in %): *TA:* Technical applicability, or part of the baseline covered by the measure. Is often 100%, but smaller if the measure is not always suitable or targets only a sub process (e.g. reducing leakage in gas transportation, but not in extraction). Values can also differ per region. *RE:* Reduction efficiency, or relative reduction of targeted emissions compared to a baseline case, averaged over multiple studies. *IP:* Implementation potential, increases in time due to increased technology diffusion and implementation and the removal of barriers. *OVcorr:* Correction for overlap. The assumption is that the least costly measures are implemented first. If a subsequent measure is applied next to one or more measures already in place, it can have a diminished benefit^1^. Note that this correction increases in time (lower value) as IP increases.

The Maximum Reduction Potential (*MRP*) (in %) in year *t* and region *r* is the combined effect of all measures:

(2) *MRP*_(*t*,*r*)_ = (*RP*_*1* (*t*,*r*)_ + *RP*_*2* (*t*,*r*)_ + *RP*_*3* (*t*,*r*)_ … + *RP*_*x* (*t*,*r*)_)∗*TP*_(*t*)_ – *Bcorr*_(*t*,*r*)_

With (all in %): *TP:* Technological progress. Increase of the reduction potential in time, as a result of new or improved technologies. *Bcorr:* Correction for emission reductions that already take place in the baseline scenario, in each region. The assumption is here that these reductions come from the least cost measures (i.e. that part of the low cost side of the MAC curve is excluded for further reductions in a mitigation scenario).

The assumption for the construction of the MAC curves is that the least costly measures are taken first. The best estimate of the costs of a specific measure was based on the average of cost estimates in literature and made regionally specific where data was available.

Marginal costs presented in literature need to be corrected for diminishing returns of measures, when multiple measures are implemented. The cost of a certain mitigation measure is based on the assumption that the measure can be fully applied to its emission source. When multiple measures are in place, the relative reduction per measure at a given cost decreases (or vice versa, the costs per reduced GHG increases). We corrected the cost of every subsequent (more expensive) measure, following:

(3) Cost new = Cost old∗1/*OVcorr*

## Footnotes

1 If measure y is aimed at reducing the same baseline emissions as measure x that is already implemented, the OVcorr of y = 1-RP_x_.
